# Supplementary material for: Prevalence, Severity and Mortality associated with COPD and Smoking in patients with COVID-19: A Rapid Systematic Review and Meta-Analysis
Source: PLoS One. 2020 May 11;15(5):e0233147. doi: 10.1371/journal.pone.0233147 (PMC7213702; doi:10.1371/journal.pone.0233147)
Supplement: S1 Appendix — (DOCX) [file pone.0233147.s001.docx]

**Prevalence, Severity and Mortality associated with COPD and Smoking in patients with COVID-19: A Rapid Systematic Review and Meta-Analysis**

Jaber S. Alqahtani MSc, ACCP^1,2^, Tope Oyelade MSc^3^, Abdulelah M. Aldhahir MSc^1,4^, Saeed M. Alghamdi MSc^5,6^, Mater Almehmadi MSc^7^, Abdullah S Alqahtani PhD^2^, Shumonta Quaderi MD^1^, Swapna Mandal MD PhD^1,8^, John R. Hurst MD PhD^1^

**Appendix:**

**Table S1: Medline search strategy.**

Database: Ovid MEDLINE(R) and Epub Ahead of Print, In-Process & Other Non-Indexed Citations and Daily <1946 to March 24, 2020>

Search Strategy:

--------------------------------------------------------------------------------

1 Coronavirus Infections/ or Coronavirus/ or COVID-19.mp.

2 novel coronavirus.mp. [mp=title, abstract, original title, name of substance word, subject heading word, floating sub-heading word, keyword heading word, organism supplementary concept word, protocol supplementary concept word, rare disease supplementary concept word, unique identifier, synonyms]

3 SARS-CoV-2.mp. [mp=title, abstract, original title, name of substance word, subject heading word, floating sub-heading word, keyword heading word, organism supplementary concept word, protocol supplementary concept word, rare disease supplementary concept word, unique identifier, synonyms]

4 2019-nCoV.mp. [mp=title, abstract, original title, name of substance word, subject heading word, floating sub-heading word, keyword heading word, organism supplementary concept word, protocol supplementary concept word, rare disease supplementary concept word, unique identifier, synonyms]

5 1 or 2 or 3 or 4

6 clinical characteristics.mp.

7 clinical characteristic*.mp. [mp=title, abstract, original title, name of substance word, subject heading word, floating sub-heading word, keyword heading word, organism supplementary concept word, protocol supplementary concept word, rare disease supplementary concept word, unique identifier, synonyms]

8 clinical features.mp.

9 clinical feature*.mp. [mp=title, abstract, original title, name of substance word, subject heading word, floating sub-heading word, keyword heading word, organism supplementary concept word, protocol supplementary concept word, rare disease supplementary concept word, unique identifier, synonyms]

10 6 or 7 or 8 or 9

11 5 and 10

**Table S2. Quality assessment:**

| First author | Population representative | Sample size  adequate | Confounders | Statistical  analysis | Missing  data | Methodology  of the  outcome | Objective  assessment | OVERALL  (0-3, higher score =  lower risk of bias) |
| --- | --- | --- | --- | --- | --- | --- | --- | --- |
| Arentz M et al. 2020 (1) | 1 | 0 | 0 | 1 | 1 | 2 | 2 | 1 |
| Guan et el. 2020 (2) | 3 | 3 | 3 | 3 | 2 | 3 | 2 | 2.7 |
| Huang Y et al. 2020 (3) | 3 | 2 | 2 | 1 | 2 | 3 | 1 | 2 |
| Huang et al. 2020 (4) | 1 | 0 | 1 | 1 | 2 | 1 | 1 | 1.1 |
| Liu et al. 2020 (5) | 1 | 1 | 2 | 1 | 0 | 1 | 1 | 1 |
| Mo et al. 2020 (6) | 3 | 2 | 3 | 3 | 2 | 3 | 2 | 2.6 |
| Wang et al. 2020 (7) | 3 | 2 | 2 | 3 | 3 | 3 | 1 | 2.4 |
| Wu et al. 2020 (8) | 2 | 1 | 2 | 3 | 3 | 3 | 2 | 2.3 |
| Wu J et al. 2020 (9) | 3 | 1 | 3 | 3 | 2 | 3 | 1 | 2.3 |
| Xu X et al. 2020 (10) | 0 | 1 | 0 | 1 | 0 | 0 | 1 | 0.4 |
| Xu XW et al. 2020 (11) | 1 | 0 | 1 | 0 | 0 | 0 | 1 | 0.4 |
| Yang et al. 2020 (12) | 3 | 2 | 3 | 3 | 2 | 3 | 1 | 2.4 |
| Zhang et al. 2020 (13) | 3 | 2 | 2 | 3 | 1 | 3 | 1 | 2.1 |
| Zhou et al. 2020 (14) | 3 | 3 | 2 | 3 | 3 | 3 | 2 | 2.7 |
| Zhu et al. 2020 (15) | 0 | 1 | 1 | 1 | 0 | 1 | 1 | 0.7 |

0 = definitely no (high risk of bias); 1 = mostly no; 2 = Mostly yes; 3 = definitely yes (low risk of bias)

**References:**

1. Arentz M, Yim E, Klaff L, Lokhandwala S, Riedo FX, Chong M, et al. Characteristics and Outcomes of 21 Critically Ill Patients With COVID-19 in Washington State. JAMA. 2020.

2. Guan WJ, Ni ZY, Hu Y, Liang WH, Ou CQ, He JX, et al. Clinical Characteristics of Coronavirus Disease 2019 in China. New England Journal of Medicine. 2020;28:28.

3. Huang Y, Tu M, Wang S, Chen S, Zhou W, Chen D, et al. Clinical characteristics of laboratory confirmed positive cases of SARS-CoV-2 infection in Wuhan, China: A retrospective single center analysis. Travel Medicine & Infectious Disease.101606.

4. Huang C, Wang Y, Li X, Ren L, Zhao J, Hu Y, et al. Clinical features of patients infected with 2019 novel coronavirus in Wuhan, China. Lancet (London, England).395(10223):497-506.

5. Liu K, Fang YY, Deng Y, Liu W, Wang MF, Ma JP, et al. Clinical characteristics of novel coronavirus cases in tertiary hospitals in Hubei Province. Chinese Medical Journal. 2020;07:07.

6. Mo P, Xing Y, Xiao Y, Deng L, Zhao Q, Wang H, et al. Clinical characteristics of refractory COVID-19 pneumonia in Wuhan, China. Clinical Infectious Diseases. 2020;16:16.

7. Wang D, Hu B, Hu C, Zhu F, Liu X, Zhang J, et al. Clinical Characteristics of 138 Hospitalized Patients With 2019 Novel Coronavirus-Infected Pneumonia in Wuhan, China. Jama. 2020;07:07.

8. Wu C, Chen X, Cai Y, Xia J, Zhou X, Xu S, et al. Risk Factors Associated With Acute Respiratory Distress Syndrome and Death in Patients With Coronavirus Disease 2019 Pneumonia in Wuhan, China. JAMA Internal Medicine. 2020;13:13.

9. Wu J, Wu X, Zeng W, Guo D, Fang Z, Chen L, et al. Chest CT Findings in Patients with Corona Virus Disease 2019 and its Relationship with Clinical Features. Investigative Radiology. 2020;21:21.

10. Xu X, Yu C, Qu J, Zhang L, Jiang S, Huang D, et al. Imaging and clinical features of patients with 2019 novel coronavirus SARS-CoV-2. European Journal of Nuclear Medicine & Molecular Imaging. 2020;28:28.

11. Xu XW, Wu XX, Jiang XG, Xu KJ, Ying LJ, Ma CL, et al. Clinical findings in a group of patients infected with the 2019 novel coronavirus (SARS-Cov-2) outside of Wuhan, China: retrospective case series. BMJ.368:m606.

12. Yang X, Yu Y, Xu J, Shu H, Xia J, Liu H, et al. Clinical course and outcomes of critically ill patients with SARS-CoV-2 pneumonia in Wuhan, China: a single-centered, retrospective, observational study. The Lancet Respiratory medicine. 2020.

13. Zhang JJ, Dong X, Cao YY, Yuan YD, Yang YB, Yan YQ, et al. Clinical characteristics of 140 patients infected with SARS-CoV-2 in Wuhan, China. Allergy. 2020;19:19.

14. Zhou F, Yu T, Du R, Fan G, Liu Y, Liu Z, et al. Clinical course and risk factors for mortality of adult inpatients with COVID-19 in Wuhan, China: a retrospective cohort study. Lancet (London, England). 2020;11:11.

15. Zhu W, Xie K, Lu H, Xu L, Zhou S, Fang S. Initial clinical features of suspected Coronavirus Disease 2019 in two emergency departments outside of Hubei, China. Journal of Medical Virology. 2020;13:13.
